# Alleviative mechanism and effect of *Bifidobacterium animalis* A6 on dextran sodium sulfate‐induced ulcerative colitis in mice

**DOI:** 10.1002/fsn3.3124

**Published:** 2022-11-09

**Authors:** Fang Wu, Guna Wuri, Bing Fang, Mengxuan Shi, Ming Zhang, Liang Zhao

**Affiliations:** ^1^ School of Food and Health Beijing Technology and Business University Beijing China; ^2^ The Innovation Centre of Food Nutrition and Human Health (Beijing) College of Food Science and Nutritional Engineering; China Agricultural University Beijing China; ^3^ Beijing Laboratory of Food Quality and Safety College of Food Science and Nutritional Engineering, China Agricultural University Beijing China; ^4^ Key Laboratory of Functional Dairy College of Food Science and Nutritional Engineering, China Agricultural University Beijing China

**Keywords:** *Bifidobacterium animalis* A6, epithelial barrier function, immune function, STAT6, ulcerative colitis

## Abstract

Probiotics have been increasingly investigated for their role in alleviating symptoms of ulcerative colitis (UC), but the specific mechanism involved remains unclear. We investigated the alleviating effect of *Bifidobacterium animalis* A6 (BAA6) in UC through a mouse dextran sulfate sodium (DSS) model. When treated with a high dose of BAA6 (1 × 10^10^ cfu/ml), it was found that colitis symptoms were significantly alleviated, and mucosal damages experienced obvious relief. Moreover, a high dose of BAA6 effectively upregulated free fatty acid receptors 2 and 3 (FFAR2 and FFAR3) expression and butyric acid metabolism specifically. Furthermore, the supplement of BAA6 significantly suppressed pro‐inflammatory cytokines levels (interleukin‐13) and the expression of pore‐forming protein claudin‐2. The upstream regulatory genes of claudin‐2, such as *STAT6*, *GATA4*, *Cdx2*, were also significantly inhibited by BAA6. Collectively, this study concludes that BAA6 attenuated DSS‐induced colitis by increasing the levels of intestinal butyric acid, activating the butyric acid‐FFAR pathway, suppressing excessive proinflammatory response, and protecting the function of the colon epithelial barrier.

## Graphical abstract

This study suggested that Bifidobacterium animalis A6 attenuated DSS‐induced colitis by increasing the levels of intestinal butyric acid, activating the butyric acid‐FFAR pathway, suppressing excessive proinflammatory response, and protecting colon epithelial barrier function.

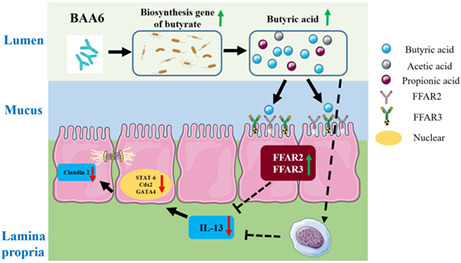

## 1 INTRODUCTION

Ulcerative colitis (UC) is the main form of inflammatory bowel disease (IBD), and the incidence of UC has been increasing worldwide for decades (Molodecky et al., 2012). UC has been convincingly shown to develop as a result of a combination of various factors, including genes, innate immunity, and environmental factors (Malmborg & Hildebrand, 2016). According to clinical studies, UC is difficult to permanently cure due to its high recurrence rate (Lakatos, 2006; Ng, Wong, & Ng, 2016). The existing drugs most commonly used to treat UC are salazosulfamide, prednisone, and antibiotics. However, the clinical uses of these drugs are hampered by adverse effects and UC's high recurrence rates (Vegas‐Sanchez, Rollan‐Landeras, Garcia‐Rodriguez, & Bolas‐Fernandez, 2015). Therefore, novel therapeutic or preventive strategies for UC are urgently needed.

Although the exact etiology and etiopathogenesis of UC have remained elusive, there is no doubt that tight junction (TJ) plays a vital role in the pathogenetic process of UC, which could connect the adjacent cells by sealing the intercellular space and regulating epithelial cell permeability (Lee, 2015). TJ proteins, which were comprised of occluding, claudins, junctional adhesion molecules, and zonula occludens, contributed to intestinal barrier function via their role in the strength and selectivity of paracellular permeability (Oshima, Miwa, & Joh, 2008). Claudin‐2 in particular has been shown to form a channel for small cations and water, resulting in increased paracellular permeability in intestinal (Luettig, Rosenthal, Barmeyer, & Schulzke, 2015). Furthermore, numerous studies have confirmed the high expression of claudin‐2 in colonic samples from UC patients (Weber, Nalle, Tretiakova, Rubin, & Turner, 2008; Zeissig et al., 2007). Claudin‐2 could form antigen complexes by binding, thus allowing the protein antigen to be internalized into epithelial cells and cause epithelial barrier dysfunction (Liu et al., 2013). Various functional food components could effectively ameliorate UC by preventing epithelial barrier dysfunction and suppressing claudin‐2 in mice (Bibi, Kang, Du, & Zhu, 2018; G. Yang, Wang, Kang, & Zhu, 2014).

Probiotics have demonstrated a protective effect in the treatment of UC, such as how administered *Bifidobacteria infantis* resulted in a therapeutic effect in dextran sodium sulfate (DSS) murine model (Ewaschuk et al., 2008). The UC patients who received treatment with a combination of *Lactobacillus* and *Bifidobacterium* showed fewer relapses and longer remission periods (Wildt, Nordgaard, Hansen, Brockmann, & Rumessen, 2011). Meanwhile, live *Lactobacillus* GG has shown improved barrier function with downregulation of claudin‐2 expression in NEC mice (Bergmann et al., 2013). Live *Bifidobacterium infantis* similarly has shown increased transepithelial resistance and decreased claudin‐2 in T84 cells (Ewaschuk et al., 2008). Probiotics may upregulate intestinal microbial metabolism of short‐chain fatty acids (SCFA), which could strengthen epithelial barrier integrity and suppress excessive inflammation response (D. Yang et al., 2021). SCFAs, like acetate, propionate, and butyrate, can activate the expression of the Free Fatty Acid Receptors (FFAR2 and FFAR3) of intestinal cells and exhibit anti‐inflammatory effects on colonic epithelium and immune cells (Kimura, Ichimura, Ohue‐Kitano, & Igarashi, 2020). Previous studies have demonstrated that the inhibition of pro‐inflammatory cytokines may contribute to the improvement of mucosal barrier function and intestinal permeability in UC patients and mice models (Creyns et al., 2020; Oh et al., 2020). These studies led us to deduce that probiotics might exhibit an ameliorating inflammatory effect via the SCFA‐FFAR pathway.

As stated above, *Bifidobacterium animalis* A6 (BAA6) was separated from the feces of the longevous population from Bama, Guangxi, China (Sun et al., 2015), which is famous for its long‐lived population and the rarity of bowel diseases among the local population. Therefore, we propose that this bacterium serve as a candidate to provide a new option for the treatment of UC. To study the possible therapeutic strategies for UC, we selected a model of oral administration of DSS induces colonic inflammation, which is similar to human UC (Pan et al., 2014; Tsang et al., 2015). The main goal of the present study was to investigate the potential effects and putative mechanism of BAA6 in the DSS colitis model.

## 2 MATERIALS AND METHODS

### 2.1 Animals and groups

Male BALB/c mice that were 6 weeks old and had a body weight of 20–25 g were obtained from Vital River (Vital River Laboratory Animal Technology Co., Ltd, Beijing, China). After quarantine, mice were divided into five groups. All experimental protocols were followed in accordance with institutional guidelines (GB 14925, 2010/63/EU). UC was induced by continuous oral administration of DSS (2.5% w/v, TdB Consultancy, Uppsala, Sweden) in distilled water from Day 1 to Day 7. The experimental protocol was as follows: Group 1, the untreated control (**Control**); Group 2, DSS‐treated group (**DSS**); Group 3, high dose BAA6 (1 × 10^10^ CFU/mL, 0.2 ml/d) treatment after 1,2‐dimethylhydrazine (DMH) initiation (**H‐BAA6/DSS**); Group 4, medium dose BAA6 (1 × 10^8^ CFU/ml, 0.2 ml/d) treatment after DMH initiation (**M‐BAA6/DSS**); Group 5, low dose BAA6 (1 × 10^6^ CFU/ml, 0.2 ml/d) treatment after DMH initiation (**L‐BAA6/DSS**). BAA6 was administered once per day to Groups 3–5 from Day 8 to Day 14.

### 2.2 Experimental design

Daily body weight was monitored. The severity of UC was assessed according to the disease activity index (DAI), which was calculated by weight loss score, fecal trait score, and blood test score as described in previous studies (Murthy et al., 1993). The mice were anesthetized and sacrificed after 14 days. Colons were then excised at necropsy at which point they were subjected to length measurements and opened longitudinally. A segment of the distal colon that was 1 cm in length was immediately fixed in PBS‐buffered 10% (v/v) formaldehyde solution. The paraffin‐embedded sections were cut for hematoxylin and eosin stain (HE) staining and immunocytochemistry. A histological score was determined as previously described (Dieleman et al., 1998).

### 2.3 Myeloperoxidase assay of colon tissue

Colonic tissues were homogenized in 9 volumes of cold saline solution. The MPO activity was determined according to the protocol described by the manufacturer (Nanjing Jiancheng Bioengineering Institute, Nanjing, China). MPO activity was reported as per gram units of sample, and one unit of MPO activity was defined as the amount of enzyme that degraded to 1 mmol hydrogen peroxide/min at 25°C.

### 2.4 Immunocytochemistry assay of colon tissue

Immunocytochemistry analyses were performed in accordance with the manufacturer's instructions (Wuhan Guge, Wuhan, China). Anti‐claudin‐2 antibodies were obtained from Abcam (Cambridge, MA, USA). Each section of the colons was deparaffinized and rehydrated, and the antigen was then retrieved. All paraffin sections were then blocked by a hydrogen peroxide/methanol solution and decolorized in PBS in order to execute the following steps. The sections were incubated with the anti‐claudin2 antibody (1:200) overnight at 4°C. After being washed with PBS, HRP‐conjugated streptavidin (Biocare Medical, Concord, CA, USA) was used as the secondary antibody and incubated with the sections for 50 min at RT. The sections were then washed in a PBS solution and coloration with 3,3‐diaminobenzidin (DAB). The yellow or brown‐yellow granules are positively stained claudin‐2 proteins. After each section was washed with distilled water to finish coloration, they were stained with hematoxylin, dehydrated, and mounted with neutral gums. The immunohistochemical staining was quantified using Image‐Pro plus software (Version 6.0, Media Cybernetics, Bethesda, MD USA) based on the previously reported protocol (Guerra et al., 2015).

### 2.5 Western blot assays

Protein expression levels of β‐actin, FFAR2, and FFAR3 were detected by WB analysis. The colon was tissue carefully removed and homogenized. After conducting a quantitative determination of protein concentration, WB analysis was performed as previously described (Araujo et al., 2017). Primary antibodies against β‐actin, FFAR2, and FFAR3 were purchased from Abcam (Cambridge, MA, USA).

### 2.6 Determination of SCFAs in the feces

Fecal samples were moderately diluted, placed at room temperature for 10 min and then centrifuged at 5000 r/min for 10 min. The supernatant was taken for 0.22 μm microporous membrane filtration for GC detection. The SCFAs content was determined and calculated according to the previously mentioned method (Ou et al., 2019). 7890B GC system (Agilent, USA) was used to determine the concentration of SCFAs.

### 2.7 Quantitative Real‐Time PCR

RNA from each colon tissue was extracted using TRIzol® Reagent (Tiangen Biotech, Beijing, China P.R.) and then reverse transcribed into cDNA. Real‐time PCR conditions were performed based on the manufacturer's protocol in the TaKaRa Real‐Time PCR Kit (Takara, Bio, Japan). The primers for Cdx2, signal transducers, and activators of transcription 6 (STAT6) and GATA4 were designed by Primer Premier (Version 5.0, Biosoft International, Palo Alto, CA). The sequences of the primers are summarized in Table 1. All reactions were performed in triplicate, and the detected mRNA expression was normalized to GAPDH.

**TABLE 1:** Primer sequences for the real‐time PCR

Gene	Sequence(5′‐3′)
*GAPDH*	F: ACCACAGTCCATGCCATCAC R: TCCACCACCCTGTTGCTGTA
*Claudin‐2*	F:CTCCTGGGATTCATTCCTGTTG R:TCAGGCACCAGTGGTGAGTAGA
*STAT6*	F:CAACGGCTCTATGTTGACTT R:AGATGCTGTTTCCCTTCC
*GATA4*	F: CTGGAGCAACCGCAAATC R: CACGAGGCAGACAAGAACTAG
*Cdx2*	F:ACAACCTTCCCAGCCTCA R:TTCTCGCAGCGTCCATAC
*Butyryl‐CoA acetate‐CoA transferase*	F: AGGATCTCGGIRTICAYWSIGARATG R: GAGGTCGTCICKRAAITYIGGRTGNGC
*Butyrate kinase*	F: TGCTGTWGTTGGWAGAGGYGGA R: GCAACIGCYTTTTGATTTAATGCATGG

Fecal bacterial DNA was extracted from fecal samples using the QIAamp® DNA Stool mini kit (Qiagen, Germany), and the DNA concentrations were then determined by Nanodrop. The abundance of butyrate kinase and butyryl CoA: acetate CoA transferase genes in DNA extracted from fecal samples was measured by real‐time PCR as described above (Xu et al., 2017). 25 ng DNA was used as a template for each reaction. The sequences of primers are summarized in Table 1. The abundance of functional genes was expressed as log_10_ gene copies of total DNA / g of fresh fecal samples.

### 2.8 Measure of Interleukin‐13 and tumor necrosis factor‐α in each group

Each of the colonic tissues were homogenized in cold Tris–HCl buffer (100 mM, pH 7.0) in the presence of protease inhibitors. After centrifugation, the protein contents of supernatant were quantified using bicinchoninic acid assay (BCA) (Beyotime, Shanghai, China). Pro‐Inflammatory cytokines (IL‐13 and TNF‐α) in supernatants were measured with the ELISA kit (R&D, Emeryville, CA, USA). The contents of cytokines were expressed as pg/mg protein.

### 2.9 Statistical analysis

Statistical analysis was performed using SPSS (Version 17.0, SPSS Inc., USA) and the results were expressed as mean ± standard deviation. An ANOVA test and unpaired Student's *t*‐test were used for evaluating the significance of the differences among the groups, with *p* value <.05 being regarded as statistically significant. The graphs were generated using GraphPad Prism (Version 8.0, GraphPad Software Inc., USA).

## 3 RESULTS

### 3.1 BAA6 alleviated colonic shortening of DSS‐treated mice

**FIGURE 1:**
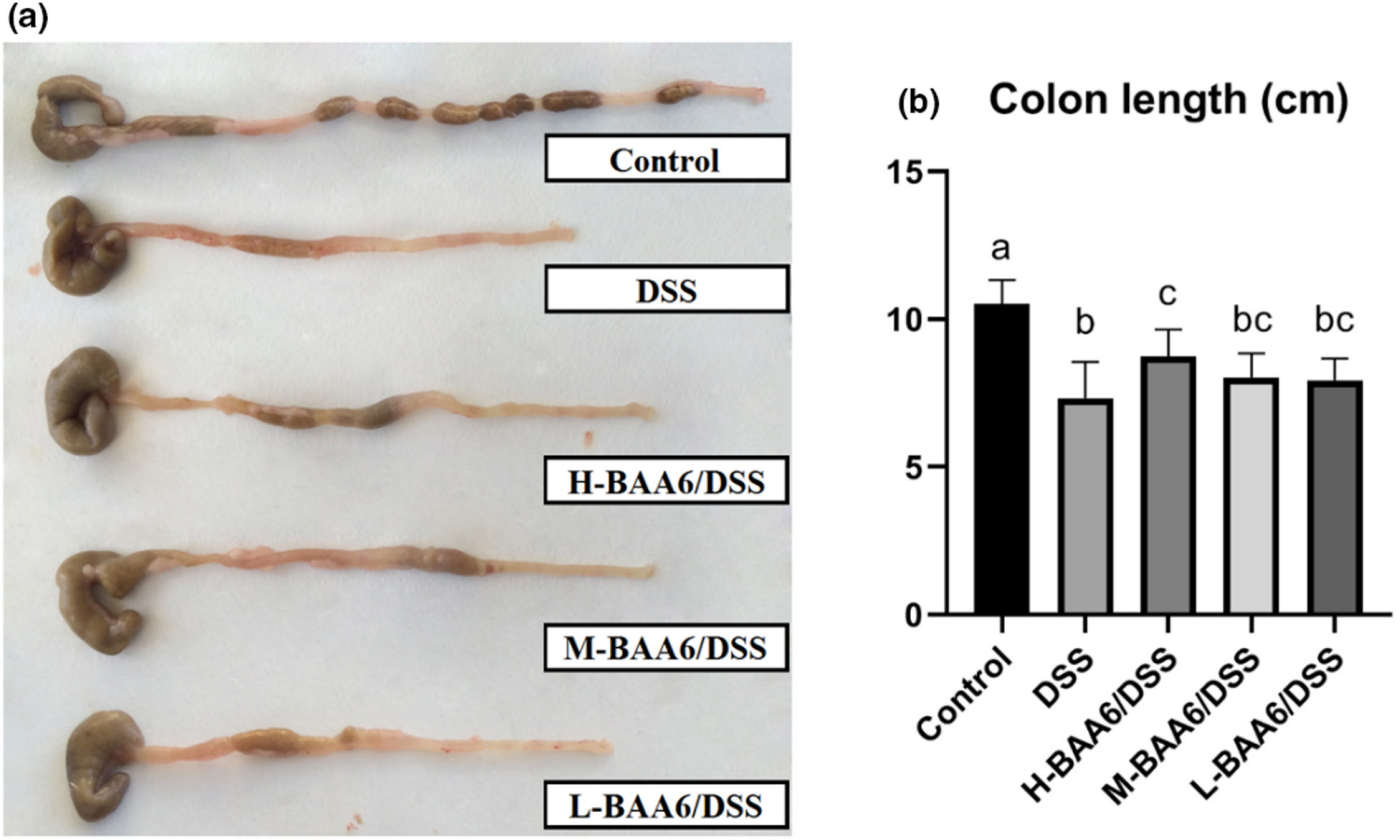
BAA6 alleviated colonic shortening of DSS‐treated mice. (a) Representative photographs of colon lengths. (b) Length of colons in all mice groups after being sacrificed.

Colonic shortening is a typical symptom of colitis in mice. As shown in Figure 1, DSS treatment shortened colon length by 30.2% compared to the control group (*p* < .05). A high dose of BAA6 (**H‐BAA6/DSS** group) significantly alleviated the effects of DSS on colon shortening (Figure 1), although it was still shorter than the control group (*p* < .05). The colon length of the **M‐BAA6/DSS** and **L‐BAA6/DSS** groups increased by 9.54% and 8.58% in comparison with the DSS group, but the increases were not noteworthy (*p* > .05).

### 3.2 BAA6 reduced the severity of colitis in mice colons

**FIGURE 2:**
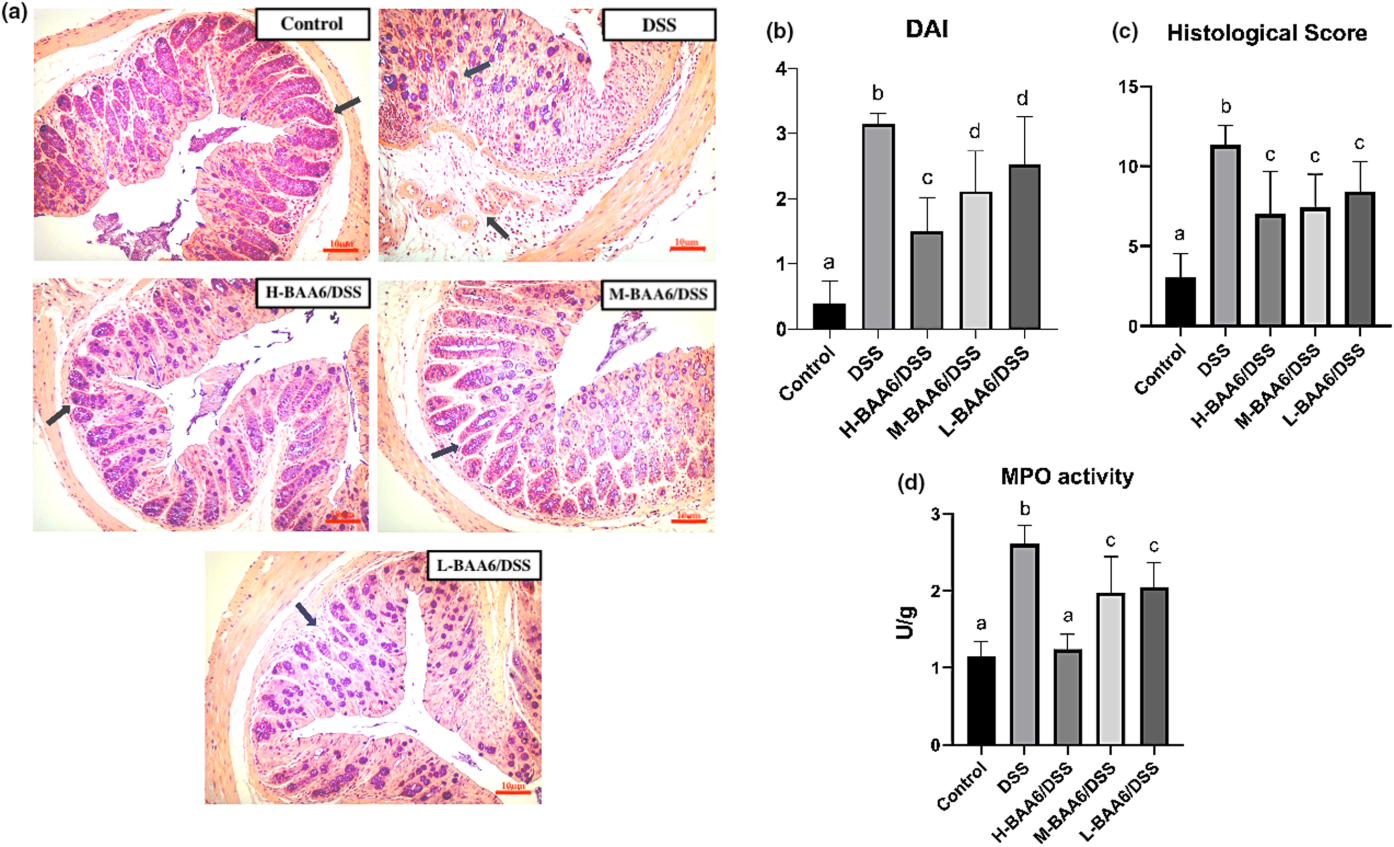
BAA6 alleviated DSS‐induced representative symptoms of colitis. (a) HE staining colonic tissue, (b) disease activity index (DAI), (c) histological scores, and (d) MPO activity.

DAI scores of different groups are summarized in Figure 2b. The DAI was much lower in mice administered with BAA6 than in DSS‐only treated mice, and it was higher compared to the control group. Significant differences between the H‐BAA6/DSS group and other groups were also observed. (*p* < .05).

The HE stainings of colon tissues are shown in Figure 2a. The colon slides of the DSS group displayed the loss of crypts, depleted goblet cells, and a destroyed epithelial layer. Oral BAA6 administration attenuated the severity of colitis in comparison with the DSS group, but they were still worse than control group. The histological scores were consistent with the HE staining observation (Figure 2c). The histological score of the administered BAA6 groups was remarkably lower than the DSS group (*p* < .05) but was not significantly different from each other.

### 3.3 BAA6 restored the upregulation of MPO activity induced by DSS

MPO activity has been reported to indirectly reflect the degree of inflammation in the tissue because it is regarded as a maker of neutrophil accumulation and activation (Tang et al., 2015). Moreover, excessive secretion of MPO in intestinal tissue occurred in response to produce oxidative stress (Sang et al., 2014). As displayed in Figure 2d, the MPO activity was significantly elevated in the DSS group in comparison with the other groups. When BAA6 was given, an obvious decrease in MPO activity was observed (*p* < .05). It is worth mentioning that no noticeable difference could be distinguished among the H‐BAA6/DSS and control group, which implies the powerful inhibitory effects on inflammation and oxidative stress of high‐dose BAA6.

### 3.4 BAA6 promoted the butyric acid metabolism of the gut microbiome

**FIGURE 3:**
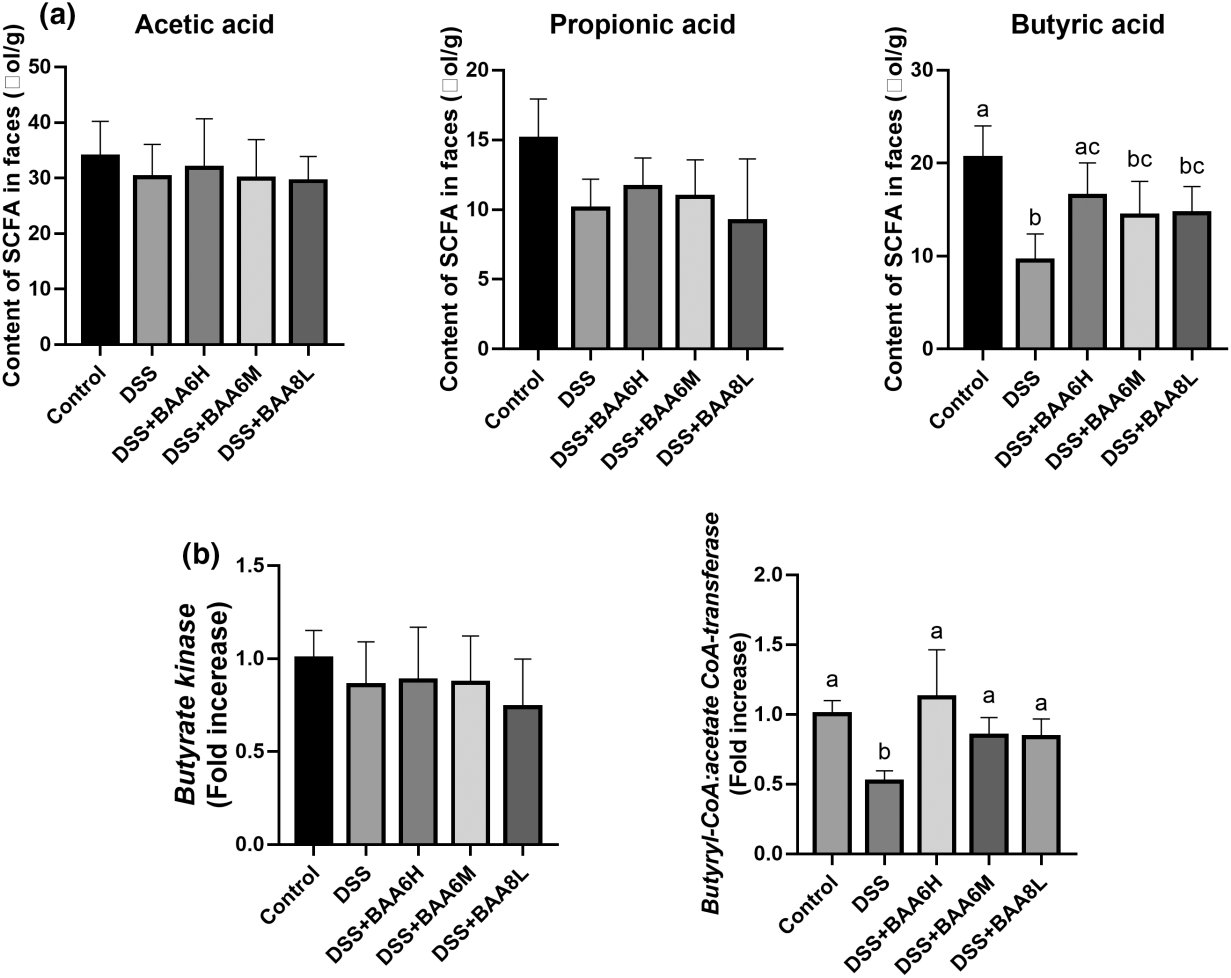
BAA6 promoted the butyric acid metabolism of gut. (a) the impact of BAA6 on fecal SCFA levels; (b) effect of BAA6 on expression levels of two major butyric acid metabolism enzymes in feces, butyrate kinase, and butyryl CoA: Acetate CoA transferase.

The impact of BAA6 on fecal SCFA levels is presented in Figure 3a. The results showed that butyric acid content in the DSS group was significantly lower than the control group (*p* < .05). At the same time, the contents of acetic acid and propionic acid in the DSS group also decreased, but the variation was not significant compared with the control group. The high doses of BAA6 significantly reversed the decrease in butyric acid induced by DSS. In addition, the rising tendencies of acetic acid and propionic acid were also observed in the BAA6 treatment group, and the change was not statically significant.

To find the cause of butyric acid upregulation, we determined the expression levels of two major butyric acid metabolism enzymes in feces, butyrate kinase, and butyryl CoA: acetate CoA transferase. As shown in Figure 3b, there were no significant differences between groups in the expression of *butyrate kinase*. However, DSS treatment significantly downregulated the expression of *butyryl CoA: acetate CoA transferase* (*p* < .05). Satisfactorily, after the intervention of BAA6 at three dosages, the levels of *butyryl CoA: acetate CoA transferase* had returned to nearly normal levels (vs. Control group, *p* > .05).

### 3.5 BAA6 activated expression levels of FFAR‐2 and FFAR‐3 in colon tissue

**FIGURE 4:**
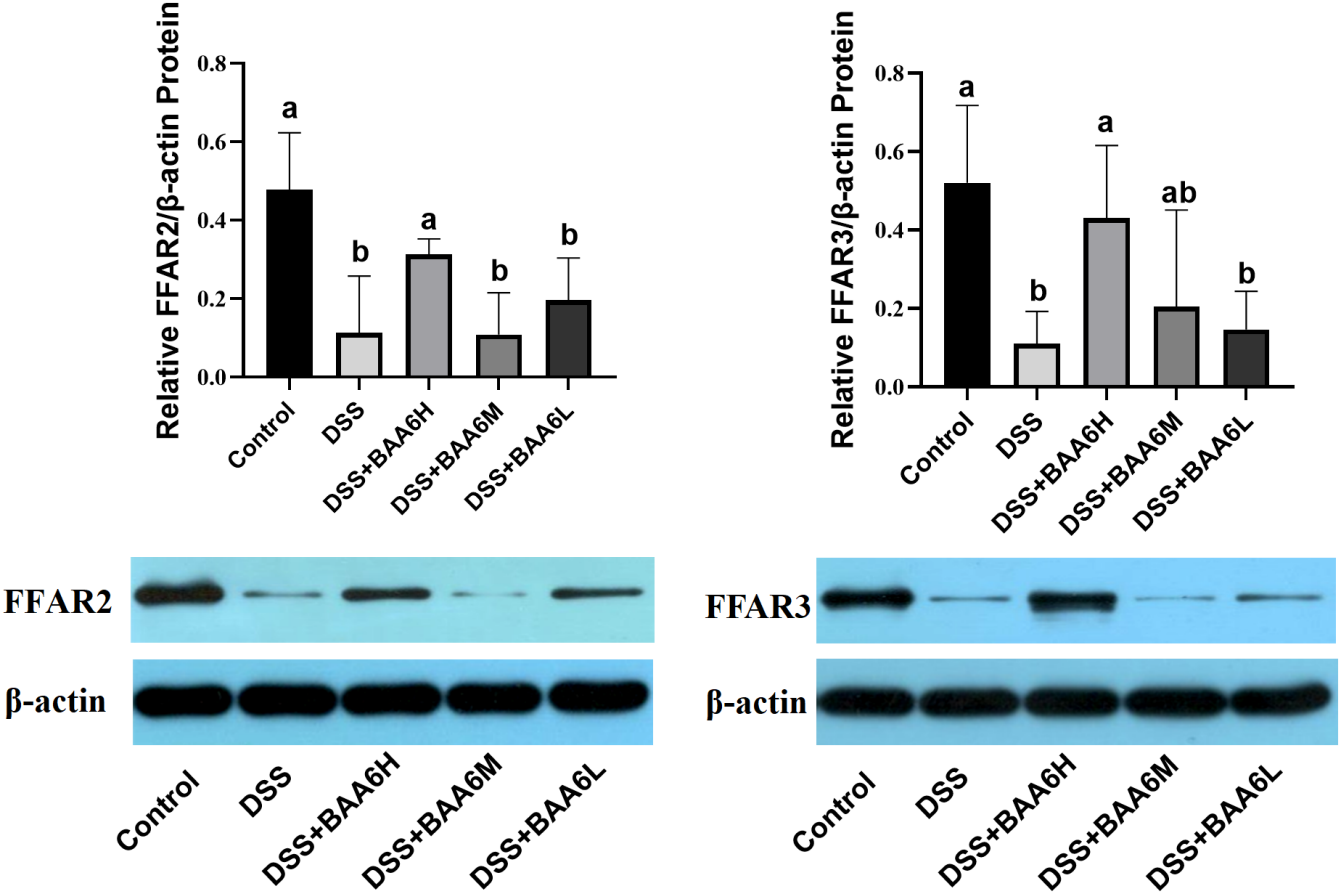
BAA6 activated protein expression levels of FFAR‐2 and FFAR‐3 in colon tissue.

FFAR2 and FFAR3 of intestinal cells can be activated by SCFAs including acetate, propionate, and butyrate. To validate whether SCFA‐FFAR activation occurred after administration of BAA6, WB analysis of FFAR was performed. As shown in Figure 4, DSS treatment significantly decreased the expression of FFAR2 and FFAR3 in colon tissue (*p* < .05). Notably, after treatment with the high dose of BAA6, the protein expressions of FFAR2 and FFAR3 were significantly increased, respectively, and were higher than in the DSS group and close to the normal levels (vs. Control group, *p* > .05). The results above implied that BAA6 may affect butyric acid metabolism and activate the expression of FFAR2 and FFAR3 in colonic mucosal cells.

### 3.6 BAA6 regulated gene expression of Claudin‐2

**FIGURE 5:**
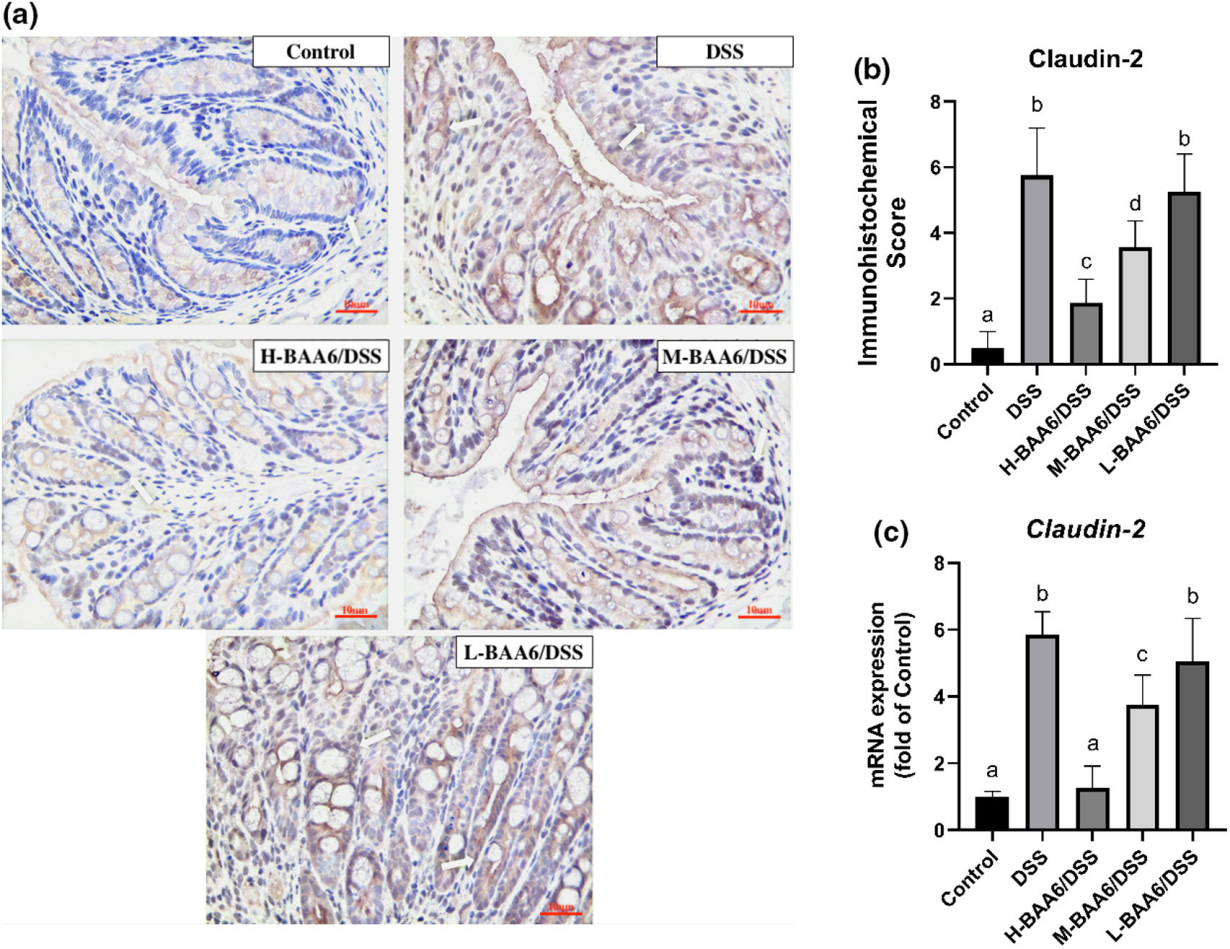
BAA6 attenuated the expression of claudin‐2 in the intestinal epithelial cells of acute colitis. (a) Representative immunohistochemical staining of claudin‐2; (b) the immunohistochemical staining score of claudin‐2; (c) *Claudin‐2* mRNA level.

Claudin‐2 is predominantly upregulated during intestinal inflammation, and it has been suggested to have channel‐forming functions that eventually lead to intestinal leakage. We thereby determined the protein and mRNA expression levels of claudin‐2 in colon tissue. Representative immunohistochemical expression of claudin‐2 in different groups is shown in Figure 5a. In normal colonic tissue, only slight immunoreactivity for claudin‐2 was observed. By contrast, claudin‐2 was highly expressed in the intestinal mucosa of DSS‐treated rats. The immunohistochemical staining score for claudin‐2 showed a similar trend (Figure 5b). The high doses of BAA6 significantly reduced claudin‐2 immunoreactivity in comparison with the DSS group (Figure 5b, *p* < .05) and eventually reinstated to the normal levels. Likewise, the RT–PCR results showed that gene expression of *Claudin‐2* was remarkably suppressed by the high and medium doses of BAA6 (Figure 5c, *p* < .05).

### 3.7 BAA6 regulated the excessive Pro‐Inflammatory response

**FIGURE 6:**
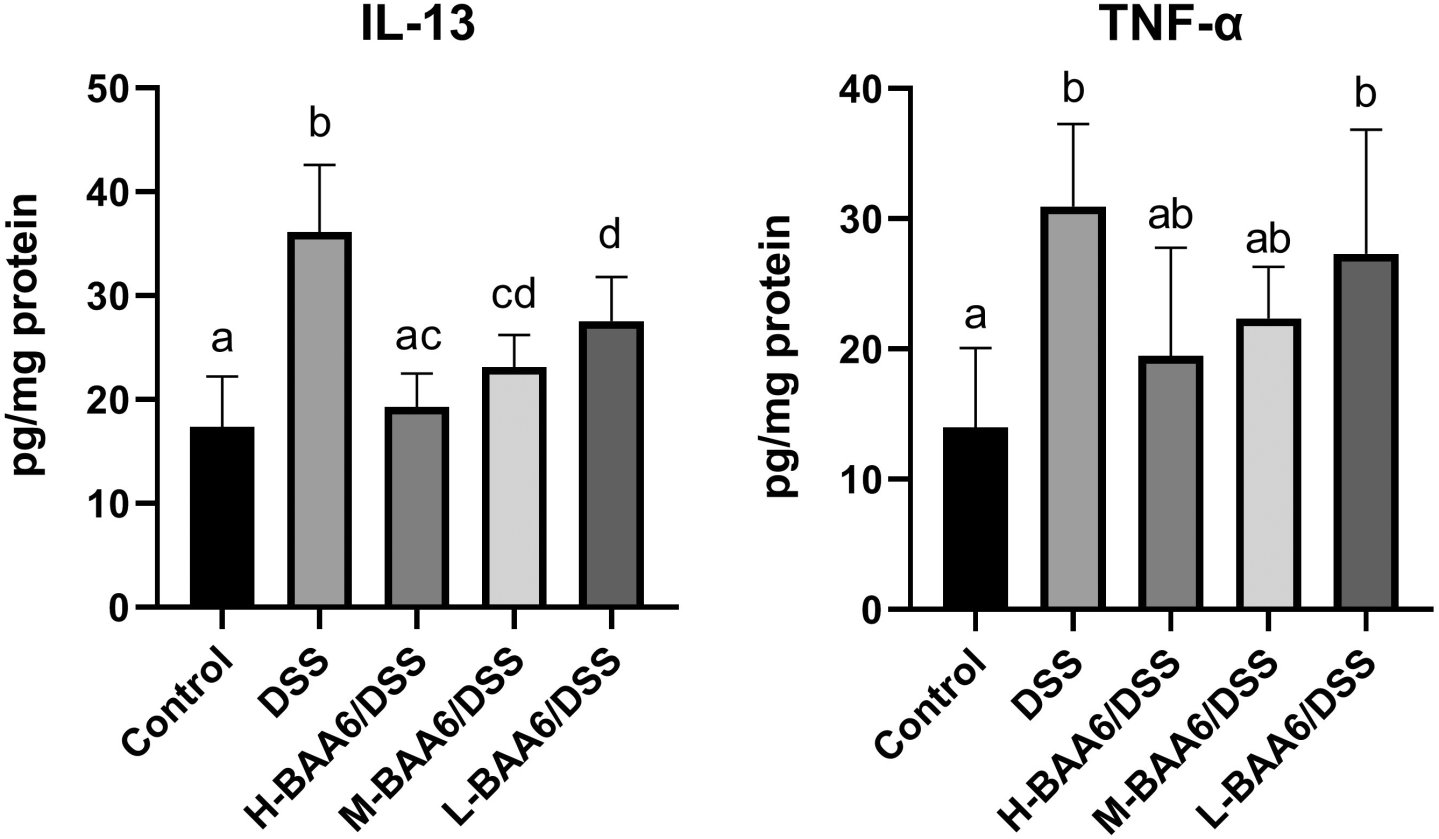
BAA6 reduced pro‐inflammatory cytokines expression in colon tissue.

Given that the upregulation of pro‐inflammatory cytokines is closely associated with claudin‐2 overexpression and FFAR deficiency (Huang et al., 2021; Rosen et al., 2011; Sakaguchi et al., 2002), we determined the expression levels of two pro‐inflammatory cytokines (TNF‐α and IL‐13). As displayed in Figure 6, IL‐13 and TNF‐α expression were significantly increased in the DSS group (*p* < .05). Oral administration of BAA6 could remarkably decrease the secretion of IL‐13 in dose‐dependent effect (*p* < .05). In comparison, the levels of TNF‐α slightly decreased after BAA6 treatment, but the reduction was not significant (*p* > .05).

### 3.8 BAA6 regulated the expression transcription factors and STAT6

**FIGURE 7:**
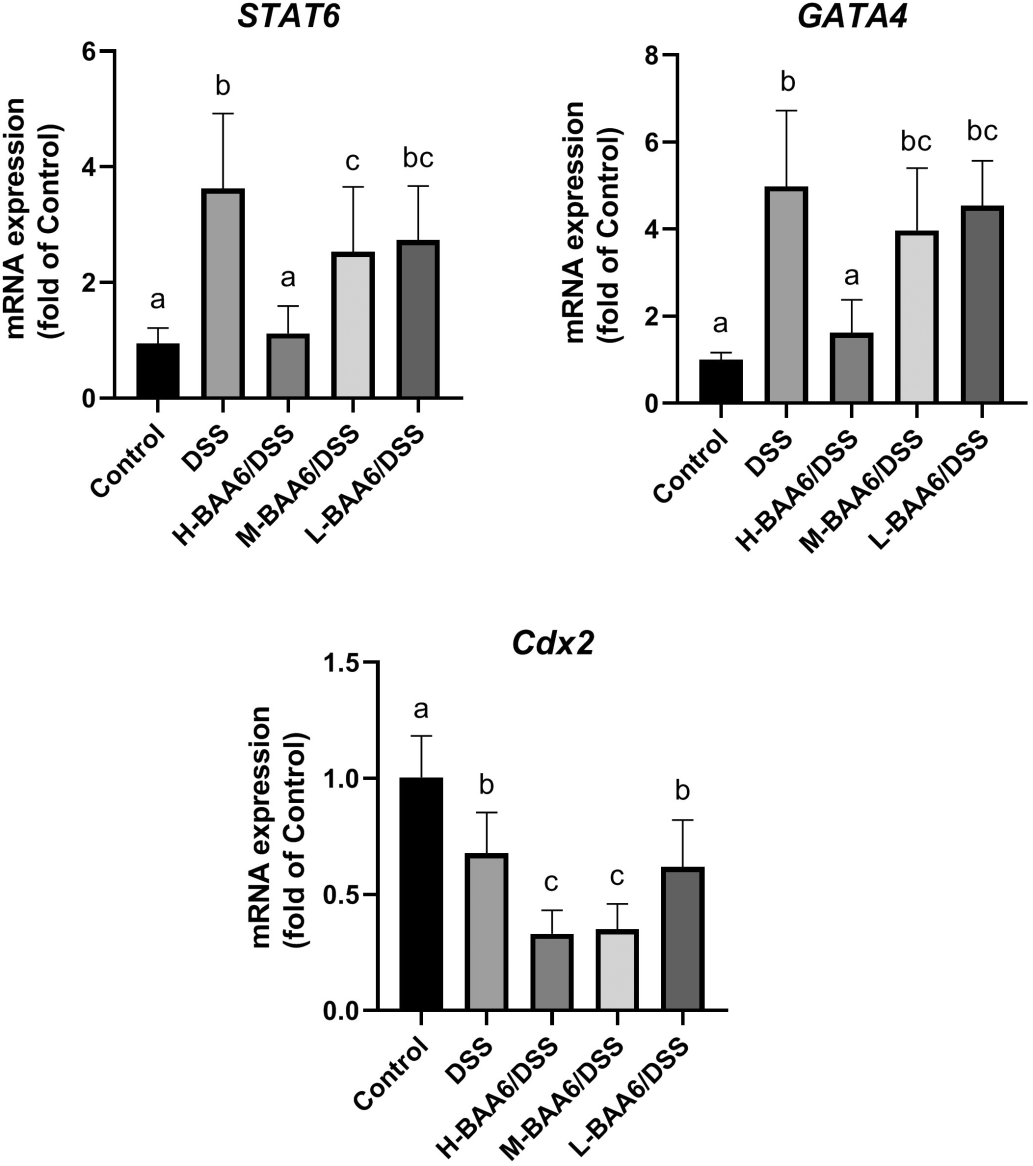
Effects of oral administration of BAA6 on *STAT6*, *GATA4*, and *Cdx2* gene expression of colon tissues.

The upregulated expression of claudin‐2 has indeed been documented and is associated with transcription factors (Cdx2, GATA4) and STAT6 (Rosen et al., 2013; Takeda, Kamanaka, Tanaka, Kishimoto, & Akira, 1996). To test the mRNA levels of STAT6, GATA4, and Cdx2, real‐time PCR analysis was performed. As shown in Figure 7a, b, the *STAT6* and *GATA4* gene expressions of the DSS group were significantly elevated in comparison with the control group (*p* < .05) and reduced in BAA6‐treated mice. The decrease was more pronounced with higher BAA6 concentrations. However, *Cdx2* gene expressions in the DSS group were slightly reduced in comparison with the control group (*p* < .05), and further decreased in the H‐BAA6/DSS and M‐BAA6/DSS groups (*p* < .05). The data collectively suggested that BAA6 supplementation inhibited the expression of Claudin‐2‐associated genes. Taken as a whole, the mRNA expression levels of the STAT6, GATA4, and Cdx2 were positively correlated with IL‐13 and claudin‐2 in each group.

## 4 DISCUSSION

UC is a complex, long‐term chronic IBD that accompanies typical symptoms such as abscesses, ulcerations, and bleeding (Di Sabatino, Biancheri, Rovedatti, MacDonald, & Corazza, 2012). Multiple studies show that probiotics might play a role in attenuating inflammation and histological symptoms in patients with UC (de Oliveira, Leite, Higuchi, Gonzaga, & Mariano, 2017). The present study illustrated BAA6 possessed preventive effects against DSS‐induced colitis via several targets. It could increase the content of SCFAs in the intestine, especially butyric acid, activate SCFA receptors (FFAR2 and FFAR3) to utilize beneficial metabolites from the gut, suppress excessive proinflammatory IL‐13 cytokines to alleviate inflammatory responses, and decrease the expression of Claudin‐2 to enhance the intestinal barrier.

The DSS‐induced colitis model effectively mimics the typical clinical and histological symptoms of human UC, such as weight loss, stool consistency, and fecal bleeding. In the mice UC model, the DAI score is used as the basis for the evaluation of these symptoms above (Pan et al., 2014). In addition, the histological score represents the injuries of intestine mucosa and muscularis, further demonstrating the severity of inflammation (Guerra et al., 2015). MPO activity has been reported to reflect the levels of inflammation in colon tissues indirectly because it is usually regarded as a maker of neutrophil accumulation and activation (Balaha, Kandeel, & Elwan, 2016; Sang et al., 2014). In this study, we proved that BAA6 played a protective role in the colitis induced by DSS as indicated by deceased DAI and the histological score, attenuated MPO activity, and mucosal damage. All these results implied that BAA6 can be used as a potential supplement to treat or prevent UC.

The content of SCFAs in feces is becoming an important clinical indicator to judge the intestinal health status of patients with IBDs, which include UC and Crohn's disease (Zhgun et al., 2020). Low levels of SCFAs may lead to a series of problems such as reduced intestinal peristalsis, barrier dysfunction, and excessive inflammation (Parada Venegas et al., 2019). Intestinal microbes, as the main producer of SCFAs, are the primary reason for the insufficient synthesis ability of SCFAs. In the present study, BAA6 intervention reversed the decrease in butyric acid induced by DSS, which suggests that BAA6 may improve the structure of intestinal microbes and the synthesis capability of butyric acid. The biosynthesis of butyrate can occur via the butyrate kinase or the butyryl CoA: acetate CoA transferase pathway (Xu et al., 2017). We also assessed the two terminal genes that provided information specifically targeting the activity of butyrate production. The present study confirmed that butyryl CoA: acetate CoA transferase upregulation may be associated with the change in butyric acid content.

SCFAs exerted beneficial physiological effects through various mechanisms, however, their signaling through FFAR2/3 is a new therapeutic target to shape host health (Mishra, Karunakar, Taraphder, & Yadav, 2020). FFAR2/3, as widely expressed cell surface receptors, can bind endogenous SCFAs specifically, which ensured that the SCFAs produced in the intestine would also be absorbed by other cells including adipocytes, splenocytes, and monocytes (Ang et al., 2016; Kimura et al., 2011; Veprik, Laufer, Weiss, Rubins, & Walker, 2016). In addition to absorption, FFAR2/3 can also be activated by SCFAs, which contributed to various biological or physiological functions such as anti‐inflammation response (Kimura et al., 2011). The present study also proved that the protein levels of FFAR‐2 and FFAR‐3 in colon tissue were upregulated after BAA6 intervention. Taken together, we speculated that administration of BAA6 promoted butyric acid biosynthesis and upregulated the expression of FFAR simultaneously, resulting in the activation of the SCFA‐FFAR signaling cascade.

The damaged intestinal barrier function is one important reason for the pathogenesis of UC (Antoni, Nuding, Wehkamp, & Stange, 2014). UC induced by DSS have been explained by a toxic effect on colonic mucous epithelial cells, followed by the stimulation of local immune responses changing the barrier function of colonic mucous epithelial cells. A damaged mucosa layer causes alterations in intercellular permeability, which is regulated by the junctional repertoire of various tight junction proteins (Fries, Belvedere, & Vetrano, 2013). To date, the tight junction protein claudin‐2 has been proven to increase the intercellular permeability of water and small cations. The over‐expression of claudin‐2 supports a key role of impaired barrier function of UC (Gunzel & Yu, 2013). Ewaschuk et al. demonstrated that *Bifidobacterium infantis* could effectively decrease expression of claudin‐2 (Ewaschuk et al., 2008). In the present study, claudin‐2 overexpression induced in the DSS group was significantly suppressed by BAA6 in protein and mRNA levels. Thus, we postulated that claudin‐2 may be the molecular target of the BAA6 anti‐inflammatory action.

Pro‐inflammatory cytokines may aggravate the severity of DSS‐induced colitis, and they were regarded as major contributing factors to mucosa barrier dysfunction. IL‐13, the key effector th2 cytokine secreted by natural killer T cells, contributed to the mucosa inflammation and claudin‐2 upregulation (Ahmad et al., 2014; Heller et al., 2005; Oshima et al., 2008). Michael J. et al. demonstrated that the mucosa barrier damage induced by IL‐13 in murine colitis is STAT6 dependent, and IL‐13 played a key role in the pathogenesis of colitis (Rosen et al., 2013). Similarly, using a STAT6 knockout mouse model, another group proved that the upregulation of intestinal permeability induced by IL‐13 is STAT6‐dependent (Rosen et al., 2011). Previous studies had proven that the IL‐13 level was markedly reduced in DSS‐induced colitis after administrated with probiotics (Mastrangeli et al., 2009; Toomer et al., 2014). However, the relationship between the STAT6 pathway and probiotics is obscure. In our study, we found that the secretion of IL‐13 was markedly inhibited in the H‐BAA6/DSS and M‐BAA6/DSS groups, which was consistent with the above reports. In addition, dietary BAA6 supplementation suppressed the *STAT6* expression level, which was proportional to the expression of claudin‐2. Based on the results of this study, BAA6 may prove to be an effective treatment in prevention against intestinal barrier dysfunction.

There was evidence that transcription factor Cdx2 and GATA4 activated the claudin‐2 promoter in the human intestinal cell line (Sakaguchi et al., 2002) considered in cooperation. Other evidences demonstrated that the protein level of claudin‐2 was upregulated by transcriptional factors (Cdx1, Cdx2, HNF‐1α, and GATA‐4) in the intestinal cell line or HIEC‐6 cells (Ikari, Sato, Watanabe, Yamazaki, & Sugatani, 2012). However, the mechanisms behind the transcription factors and probiotics are rarely the focus of research. In the present study, *GATA4* expression levels after DSS treated were prominently increased in comparison with the control group, which were significantly attenuated by BAA6 supplementation. In addition, the *Cdx2* expression levels were inhibited by BAA6 supplementation. These results suggest that *GATA4* and *Cdx2* genes have been implicated in regulating the BAA6 protective processes. Collectively, we concluded that the main alleviating mechanism of BAA6 was improving intestinal epithelial barrier function through the inhibition of claudin‐2, which was regulated by the suppression of the IL‐13‐induced STAT6 pathway.

## 5 CONCLUSIONS

In summary, the present study illustrated that BAA6 possessed preventive effects against DSS‐induced colitis via several targets. It could increase the content of SCFAs in the intestine, especially butyric acid, and activate SCFA receptors (FFAR2 and FFAR3) to utilize beneficial metabolites from gut. Meanwhile, supplementing BAA6 significantly suppressed pro‐inflammatory cytokines levels (interleukin‐13) and the expression of pore‐forming protein claudin‐2. In addition, upstream regulatory genes of claudin‐2, such as *STAT6*, *GATA4*, and *Cdx2*, were significantly inhibited by BAA6. Overall results suggest that BAA6 could be explored as a promising prophylactic agent for UC.

## FUNDING INFORMATION

This work was supported by the National Natural Science Foundation of China (31601443, 32101938).

## CONFLICT OF INTEREST

The authors declare no conflict of interest.

## Data Availability

The data that support the findings of this study are available on request from the corresponding author.
